# Systematic Review of Fatigue in Individuals With Cerebral Palsy

**DOI:** 10.3389/fnhum.2021.598800

**Published:** 2021-03-15

**Authors:** Luca Puce, Ilaria Pallecchi, Karim Chamari, Lucio Marinelli, Tiziano Innocenti, Riccardo Pedrini, Laura Mori, Carlo Trompetto

**Affiliations:** ^1^Department of Neuroscience, Rehabilitation, Ophthalmology, Genetics, Maternal and Child Health, University of Genova, Genoa, Italy; ^2^CNR National Research Council, SPIN Institute, Department of Physics, Genoa, Italy; ^3^ASPETAR, Orthopedic and Sports Medicine Hospital, Doha, Qatar; ^4^Laboratory “Sport Performance Optimization”, National Center of Sports Medicine and Sports Sciences Centre national de médecine et des sciences du sport (CNMSS), Institut supérieur du sport et éducation physique (ISSEP) Ksar-Said Manouba University, Den Den, Tunisia; ^5^IRCCS Ospedale Policlinico San Martino, Genoa, Italy; ^6^Department of Health Science, Faculty of Science, Vrije Universiteit Amsterdam, Amsterdam Movement Sciences, Amsterdam, Netherlands

**Keywords:** cerebral palsy, self-reported fatigue, objective fatigue, muscle fibers, Paralympic athletes

## Abstract

In this systematic review, we collected and analyzed literature works comparing self-reported fatigue and objectively-measured fatigue in individuals with cerebral palsy (CP) and in age-matched typically developing/typically developed (TD) controls (Healthy). The search was conducted on four electronic databases/platforms (PubMed, Web of Science, Cochrane Library, and Scopus) using the key words “cerebral palsy” combined with “fatig^*^,” where the asterisk was used as a wildcard. As a critical appraisal tool, the Joanna Briggs Institute Critical Appraisal Checklist for Quasi-Experimental Studies (2017) was used. A total of 22 studies passed the critical appraisal rating and were included in both narrative and quantitative analyses. The overall evidence quality of the findings was considered very good. Data of objectively-measured fatigue in performing maximal fatiguing tasks indicated lower fatigue levels in participants with CP, possibly due to their pathological inability to recruit highly fatigable muscle fibers. Highly trained individuals with CP and TD controls performing maximal fatiguing tasks seem to be an exception to this, as they exhibited similar levels of fatigue. In submaximal fatiguing tasks, including daily physical activities, either objectively-measured or self-reported fatigue was higher in participants with CP than in TD controls, indicating a lower ability for development of neurophysiological compensation for fatigue among participants with CP. Further studies on fatigue are needed to gain an insight into the multifold mechanisms of fatigue in individuals with CP. Understanding fatigue mechanisms could help in setting up strategies for effective intervention programs, with benefits in healthcare and improved quality of life of individuals with CP.

**Systematic Review Registration:** [PROSPERO 2019], identifier [CRD42019143524].

## Introduction

CP is defined as a group of disorders of movement and posture caused by a non-progressive lesion in the developing brain (Rosenbaum et al., 2007b). CP is one of the causes of the Upper Motor Neuron Syndrome (UMNS). The more disabling UMNS phenomenon is muscle weakness. Other UMNS phenomena include spasticity, spastic dystonia, co-contraction, and muscle spasms (Trompetto et al., 2019). In individuals with CP, UMNS is often accompanied by movement disorders, due to impairment of the basal ganglia and motor ataxia (Oskoui et al., 2013). Furthermore, disturbances of sensation, perception, cognition, communication, and behavior are often reported (Rosenbaum et al., 2007a).

No clear and widely accepted definition of fatigue is available. It can be divided into physiological fatigue and psychological/subjective fatigue. As far as the motor aspects of physiological fatigue are concerned, fatigue was defined as “*a reduction in force output that occurs during sustained voluntary activity”* (Bigland-Ritchie et al., 1983), and more recently updated as “*a transient reduction in the capacity to generate maximal force after exercise”* (Gandevia, 2001). Physiological fatigue includes two different types of fatigue, peripheral and central fatigue, indeed fatigue may occur at any point along the activation process from the cortex to the muscle. Peripheral fatigue occurs due to processes that are distally located with respect to the neuromuscular junction. Its mechanisms are related to ionic and metabolic changes in the muscle fibers, causing impaired action potential propagation and excitation-contraction coupling (Kirkendall, 1990). Central fatigue was defined as “*the inability of the brain to maintain the drive necessary to produce the desired force or power output”* (Gandevia, 2001; Allen et al., 2008). However, the boundary between peripheral and central fatigue is blurred.

While the above definitions refer to the objective motor aspects related to physiological fatigue, the psychological and subjective character of fatigue was better described as “*a reduced capacity to perform multiple tasks over time, and the experience of feeling exhausted, tired, weak, or lacking energy”* (Kaasa et al., 1999). How physiological fatigue relate to psychological/subjective fatigue is not evident in general.

Although fatigue may greatly impact daily functioning and locomotion capacity in individuals with CP (Van der Slot et al., 2012), based on the relevant literature it is not easy to understand if fatigue in individuals with CP is higher (Jahnsen et al., 2003; Opheim et al., 2009; Leunkeu et al., 2010; Maanum et al., 2010; Nieuwenhuijsen et al., 2011; Van der Slot et al., 2012; Slaman et al., 2013; Eken et al., 2014, 2016, 2017, 2019; Russchen et al., 2014; Vitiello et al., 2016; Lundh et al., 2018) lower, (Moreau et al., 2008, 2016; Eken et al., 2013; Neyroud et al., 2017), or otherwise comparable (Stackhouse et al., 2005; Doix et al., 2013; Runciman et al., 2015, 2016) with respect to fatigue in TD controls.

**Figure 1:**
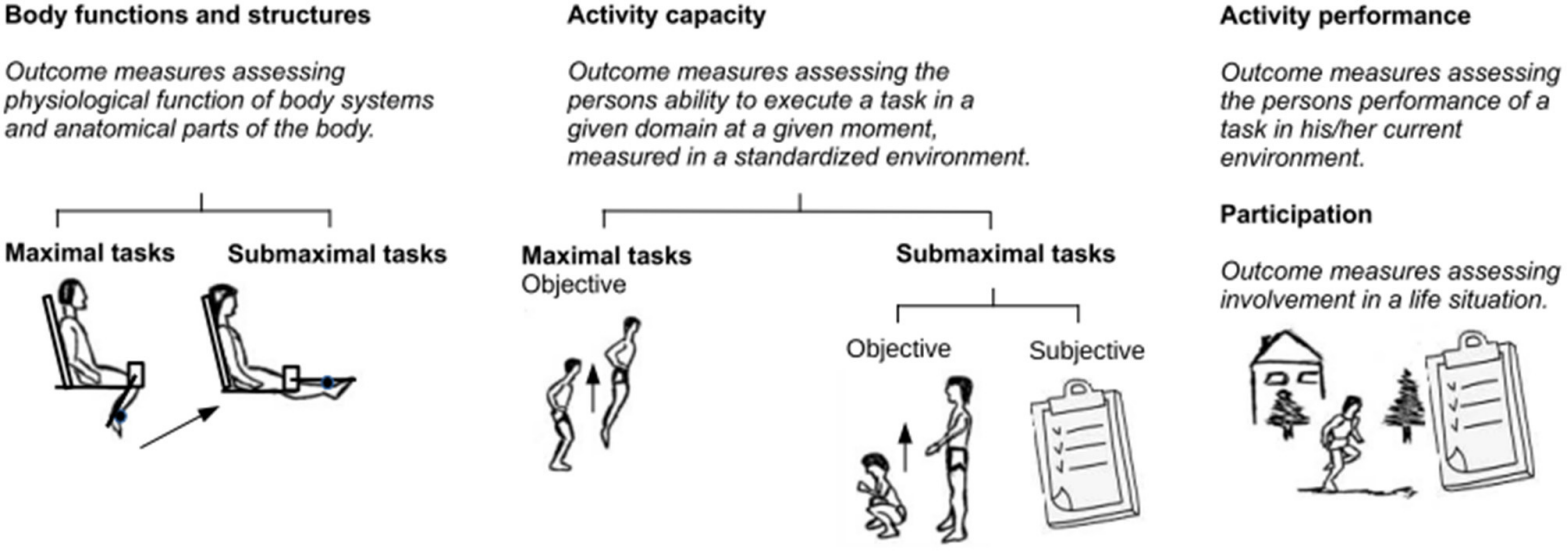
Fatigue outcome measures classified according to International Classification of Functioning (ICF) levels.

The variety of literature results and the above-mentioned apparent inconsistencies should be viewed considering the different types of measured outcomes and the specific protocol of the fatiguing tasks used in each study. Within the framework of the International Classification of Functioning, Disability and Health (ICF) (Battistella and Moran de Brito, 2002), outcome measures to assess fatigue can be classified according to three levels: “*body functions and structures*,” “*activity*,” and “*participation*” (Figure 1). Outcome measures at the “*body functions and structures*” level assess fatigue in a single muscle or in a group of muscles (i.e., an anatomical part of the body), mostly through evaluation of torque and parameters of electromyography (EMG). Outcome measures at the “*activity*” level investigate the impact of fatigue in performing complex motor tasks, such as squatting, walking, running, and cycling. In this class, fatigue can be measured both objectively or subjectively as the decrease in the performance of complex motor tasks, which can be carried out either in a laboratory setting (“*capacity*”) or in an environmental setting (“*performance*”). Finally, outcome measures at the “*participation*” level assess the restrictions that an individual may experience in involvement in life situations. At this level, only subjective fatigue is measured.

The question addressed by this systematic review is whether the above-mentioned apparent inconsistency in literature results on fatigue in individuals with CP when compared to TD people can be explained either by the different types of measured outcomes, by the specific protocol of the fatiguing tasks, or by both of these.

We believe that the knowledge of the degree of fatigue experienced by individuals with CP in performing different tasks could be very useful for designing more effective, adaptive, and safe rehabilitation programs and training methods for individuals with CP.

## Methods

### Protocol and Registration

The reporting of this systematic review was performed following the Preferred Reporting Items for Systematic Reviews and Meta-Analyses (PRISMA) Checklist. Methods of the analysis and inclusion criteria were specified in advance and documented in a protocol (PROSPERO 2019 registration number CRD42019143524).

### Information Sources

Studies were identified by searches conducted on four electronic databases/platforms (Medline by PubMed, Web of Science, Cochrane Library-CENTRAL, and Scopus). Further searches were carried out by visually scanning reference lists of articles and by using the “similar articles” option of PubMed.

### Search

Searches were conducted until August 19th, 2019 by two authors independently. Disagreements between the authors were resolved by a consensus-based discussion, followed by consultation with a third review author. The following combination of key words was searched for: “cerebral palsy,” “fatig^*^,” where ^*^ referred to a wildcard. More specifically, below are the details of the search strategy:

1. PubMed. “cerebral palsy” in [ALL] AND “fatig^*^” in [ALL];
2. Web of Science. “cerebral palsy” in [ALL FIELDS] AND “fatig^*^” in [ALL FIELDS];
3. Cochrane Library Central. “cerebral palsy” in [Title Abstract Keyword] AND “fatig^*^” in [Title Abstract Keyword];
4. Scopus. “cerebral” AND “palsy” in [TITLE-ABS-KEY] AND “fatig^*^” in [TITLE-ABS-KEY].

### Study Selection

Screenings at multiple successive stages were carried out by two authors (LP and IP) independently on the basis of (1) title, (2) abstract reading, and (3) full-text reading. Disagreements between the two authors were resolved by either consensus or consultation with a third review author.

### Eligibility criteria

#### Types of Studies

Among the types of study designs, quasi-experimental studies were considered. Restrictions of eligibility for the review were based on publication status (published), language (English), and type of publication (journal article). The time interval for eligible studies was set from any date up to August 19th, 2019.

#### Types of Participants

We selected studies where the addressed population consisted of individuals with CP. There were no restrictions on the age and sex of the study participants. All levels of functional limitation (Gross Motor Function Classification System-GMFCS I-V) were considered. Among studies reporting objective measurements of fatigue, the prerequisites for inclusion were that the individuals with CP: (1) should not have undergone orthopedic surgery within 12 months prior to testing; (2) should not have received botulinum toxin injections in testing related muscles within 6 months prior to testing. Among studies reporting either subjective or objective measurements of fatigue, the prerequisite for inclusion was that the individuals with CP: were not to have reported comorbidities impacting physical activities. Specific *post-hoc* qualitative analysis was done on highly trained individuals with CP.

As comparators, we considered TD populations, matched in terms of relevant age range. Exceptions were allowed for some self-perceived fatigue studies (Jahnsen et al., 2003; Opheim et al., 2009; Maanum et al., 2010; Nieuwenhuijsen et al., 2011; Van der Slot et al., 2012; Slaman et al., 2013; Russchen et al., 2014; Lundh et al., 2018) using as reference the scores of the general population to the same questionnaires, published in widely recognized studies (Loge et al., 1998; Merkies et al., 1999; Minderhoud et al., 2003; Lerdal et al., 2005; Valko et al., 2008).

#### Types of Intervention

We considered the following types of tasks as fatigue:

1. voluntary concentric and/or eccentric isokinetic muscle contractions, concentric and/or eccentric isotonic muscle contractions, isometric muscle contractions;
2. involuntary (electrically elicited) muscle contractions;
3. functional activities such as walking, running, performing series of squats, cycling, and swimming;
4. daily physical activities.

#### Types of Outcome Measures

The included studies considered the following outcome measures for fatigue:

1. The ICF level of “body functions and structures” considered:
  1a) force or torque output
  1b) EMG median frequency
  1c) EMG amplitude
2. The ICF level of “activity,” “capacity” sublevel considered:
  2a) performance in a motor task such as squatting, walking, running, and cycling, carried out in a laboratory setting
  2b) self-perceived fatigue in a motor task in a laboratory setting
3. The ICF level of “activity,” “performance” sublevel considered:
  3a) self-perceived fatigue in a motor task carried out in an environmental setting
4. The ICF level of “participation” considered:
  4a) self-perceived fatigue in life situations

Regarding the objective methods at the level of “body functions and structures,” the most used outcome measure is the evaluation of torque. Furthermore, EMG is the other common method used to assess fatigue through the evaluation of the median frequency and the amplitude of the signal. The shift of the median frequency toward lower values is considered a direct measure of fatigue (Komi and Tesch, 1979). As far as EMG amplitude is concerned, in submaximal fatiguing tasks, increase in EMG amplitude is dependent on the mechanisms aimed at compensating for fatigue. On the other hand, in maximal fatiguing tasks, decrease in EMG amplitude is a direct measure of fatigue (Merletti et al., 2004).

### Data Collection Process

In order to sort out the studies and extract data, a data sheet form was developed, containing the following sections: first author and year of publication, study design, characteristics of the participants (number, diagnosis, age, sex, weight, height, spasticity, GMFCS, etc.), type of fatiguing task (ICF level, maximal/submaximal fatiguing task), type of fatigue (objective/subjective), outcome measures, muscles under observation, main results, and quantitative results.

Collection of quantitative data was done by retrieving data found in the selected studies in the form of either numerical data or data points in plots. In the latter case, numerical data were extracted by digitalization of the plots.

The data extraction and filling of the data sheet form was done by two authors alternately (LP and IP), with a mutual check on each entry. Disagreements were resolved by either consensus or consultation with a third review author.

### Assessment of Methodological Quality

Eligible studies were critically appraised by two independent reviewers (LP and IP) using the Joanna Briggs Institute (JBI) Critical Appraisal Checklist for Quasi-Experimental Studies (JBI, 2020).

A cut-off point of minimum 50% “yes” scores was set on the JBI critical assessment tool as a criterion for inclusion of studies in the review (Moola et al., 2020).

### Summary Measures and Methods of Analysis

Narrative synthesis and synoptic tables were conducted as a primary mechanism of qualitative data synthesis, while further quantitative analysis was conducted for the studies where data were available. The latter quantitative analysis collected data of differences between the mean change of responses in individuals with CP and control groups, where the change occurred during the fatiguing task. As these were continuous data, the standardized mean differences (SMDs) of the outcomes were calculated as the ratios of mean differences between the responses in individuals with CP and control groups, μ_1_ and μ_2_ respectively, to the pooled standard deviation σ_pooled_. SMD data provided an indication of the direction and magnitude of fatigue in individuals with CP as compared to TD controls. Forest plots were then developed as visual representations of the direction and magnitude of the effect, allowing a comparison of measurements carried out with different methods in homogeneous subgroups of studies. Attention was paid on the sign used to denote the measurement (direction of the effect) carried out with different methods. Data were plotted in such a way that highly positive values indicated a larger amount of fatigue in individuals with CP.

The possibility of carrying out meta-analysis of data, consisting of measurement of the overall effect size within the subgroups of studies, was considered. The appropriateness of conducting a quantitative meta-analysis was judged firstly in terms of clinical and methodological homogeneity, and secondarily in terms of statistical homogeneity. Statistical homogeneity of different studies (Higgins and Thompson, 2002) was evaluated using the I-square statistic (*I*^2^) test, with a threshold *I*^2^ ≤ 56%.

## Results

### Selection of Studies

**Figure 2:**
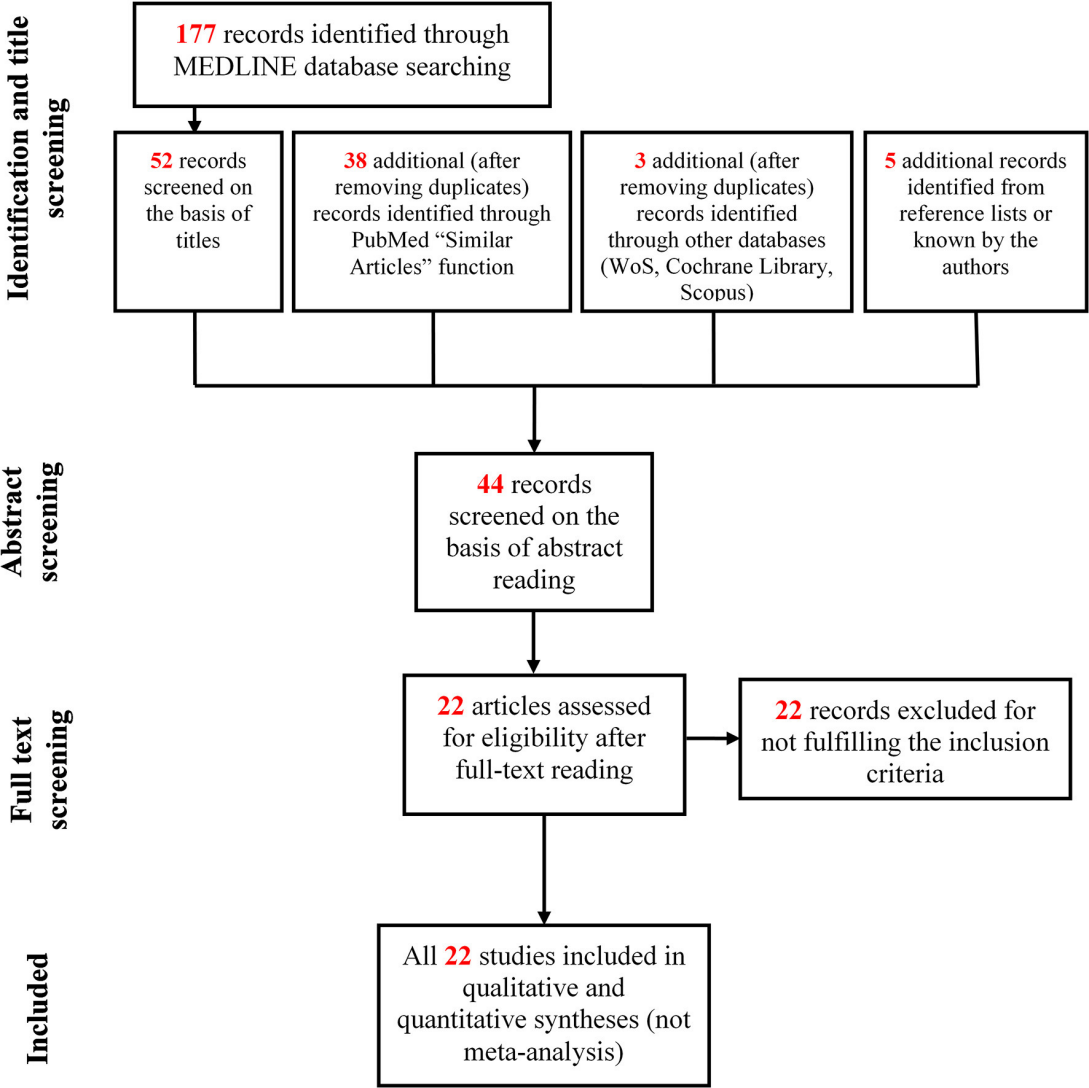
Flow chart of study selection.

Below, we have listed the chronological developments and the outcomes of the search process in detail. This process is also summarized in the flow chart of Figure 2.

1. Platform: PubMed; search combination: (cerebral palsy) [ALL] AND fatig^*^ [ALL]; date of the last search: 13/05/2019; number of results: 177; number of results after title screening: 52.
2. Platform: PubMed; additional results retrieved using the “similar articles” option of PubMed from the results of (1): 28; dates of the search: 14/05/2019 – 15/05/2019.
3. Database: Web of Science; search combination: ALL FIELDS: (cerebral palsy) AND ALL FIELDS: (fatig^*^); date of the last search: 15/05/2019; number of results: 238; number of results after title screening and after removing duplicates from (1) and (2) searches: 2.
4. Platform: PubMed; additional results retrieved using the “similar articles” option of PubMed from the results of (3): 10; date of the search: 16/05/2019.
5. Database: Cochrane Library (CENTRAL); search combination: cerebral palsy in Title Abstract Keyword AND fatig^*^ in Title Abstract Keyword; date of the last search: 16/05/2019; number of results: 90; number of results after title screening and after removing duplicates from (1) to (4) searches: 1.
6. Database: Scopus; search combination: [TITLE-ABS-KEY (cerebral AND palsy) AND TITLE-ABS-KEY (fatig^*^)]; date of the last search: 16/05/2019; number of results: 406; number of results after title screening and after removing duplicates from (1) to (5) searches: 0.
7. Scanning of reference lists of articles; number of results after removing duplicates from (1) to (6) searches: 5.

The total number of results from (1) to (7) was 98.

The total number of results after successive abstract screening was 44.

The results of the successive full-text reading were 22.

### Methodological Quality

The appraisal results for the included studies are outlined in Table 1. All 22 studies included in the review scored more than 50% “yes” on the critical assessment checklist. Only one study required a follow-up (Opheim et al., 2009). Six studies did not have a control group (Jahnsen et al., 2003; Opheim et al., 2009; Nieuwenhuijsen et al., 2011; Van der Slot et al., 2012; Slaman et al., 2013; Russchen et al., 2014). Similarity of participants included in any comparison was unclear in two studies (Moreau et al., 2016; Eken et al., 2017). Reliability of the method of outcome measurements was unclear in two studies (Maanum et al., 2010; Runciman et al., 2016). In six studies, presence of multiple measurements of the outcome both pre- and post-intervention/exposure was not applicable (Jahnsen et al., 2003; Opheim et al., 2009; Nieuwenhuijsen et al., 2011; Van der Slot et al., 2012; Slaman et al., 2013; Russchen et al., 2014). According to the JBI systematic review guideline, the overall evidence quality of the findings was considered very good.

**Table 1:** Critical appraisal ratings of selected Quasi-Experimental Studies.

**References**	**Q1**	**Q2**	**Q3**	**Q4**	**Q5**	**Q6**	**Q7**	**Q8**	**Q9**	**%**
Doix et al. (2013)	•	•	•	•	•	•	•	•	•	90
Eken et al. (2013)	•	•	•	•	•	•	•	•	•	90
Eken et al. (2014)	•	•	•	•	•	•	•	•	•	90
Eken et al. (2016)	•	•	•	•	•	•	•	•	•	90
Eken et al. (2017)	•	•	•	•	•	•	•	•	•	80
Eken et al. (2019)	•	•	•	•	•	•	•	•	•	90
Jahnsen et al. (2003)	•	•	•	•	•	•	•	•	•	70
Leunkeu et al. (2010)	•	•	•	•	•	•	•	•	•	90
Lundh et al. (2018)	•	•	•	•	•	•	•	•	•	90
Maanum et al. (2010)	•	•	•	•	•	•	•	•	•	80
Moreau et al. (2008)	•	•	•	•	•	•	•	•	•	90
Moreau et al. (2016)	•	•	•	•	•	•	•	•	•	80
Neyroud et al. (2017)	•	•	•	•	•	•	•	•	•	90
Nieuwenhuijsen et al. (2011)	•	•	•	•	•	•	•	•	•	70
Opheim et al. (2009)	•	•	•	•	•	•	•	•	•	80
Runciman et al. (2015)	•	•	•	•	•	•	•	•	•	90
Runciman et al. (2016)	•	•	•	•	•	•	•	•	•	80
Russchen et al. (2014)	•	•	•	•	•	•	•	•	•	70
Slaman et al. (2013)	•	•	•	•	•	•	•	•	•	70
Stackhouse et al. (2005)	•	•	•	•	•	•	•	•	•	90
Van der Slot et al. (2012)	•	•	•	•	•	•	•	•	•	70
Vitiello et al. (2016)	•	•	•	•	•	•	•	•	•	90

JBI Methodological quality appraisal Checklist to be score as “Yes•, No•, Unclear•, Not/Applicable•”. Q1. Is it clear in the study what is the ‘cause' and what is the ‘effect' i.e. there is no confusion about which variable comes first. Q2. Were the participants included in any comparisons similar? Q3. Were the participants included in any comparisons receiving similar treatment/care, other than the exposure or intervention of interest? Q4. Was there a control group? Q5. Were there multiple measurements of the outcome both pre and post the intervention/exposure? Q6. Was follow up complete and if not, were differences between groups in terms of their follow up adequately described and analyzed? Q7. Were the outcomes of participants included in any comparisons measured in the same way? Q8. Were outcomes measured in a reliable way? Q9. Was appropriate statistical analysis used?

### Outcome Measures at “Body Function and Structures” Level

A total of 13 studies were included at the “body function and structures” level, out of which two studies are described separately in section outcome measures at “body function and structures” levels and “capacity” levels in maximal fatiguing tasks in highly trained athletes. Among the remaining 11 studies, four studies used a maximal fatiguing task, while the others used a submaximal fatiguing task.

#### Maximal Fatiguing Tasks

**Figure 3:**
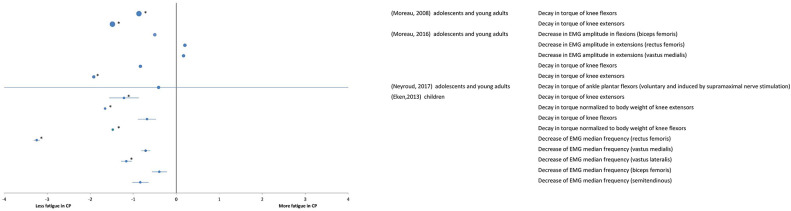
Forest plot of fatigue data in maximal tasks extracted from “body functions and structures” measures. The size of the symbols is proportional to the number of participants. The horizontal bars indicate the 95% confidence interval. * indicates statistical significance between the groups (*p* < 0.05).

A total of 4 studies at the “body function and structures” level used a maximal fatiguing task, and their results are reported as SMDs in the forest plot of Figure 3. Study details and results are also summarized in Table 2. The participants in the study (Eken et al., 2013) were children whereas the participants in the studies by Moreau et al. (2008, 2016), Neyroud et al. (2017) were adolescents and young adults. The overall effect size was not calculated due to heterogeneity of data in terms of participant characteristics and the muscles under investigation. In studies by Eken et al. (2013) and Moreau et al. (2016), the rate of decline in peak torque maximal isokinetic knee extensions was significantly smaller in the individuals with CP compared with that of the TD participants, implying more fatigue in the latter ones. However, during knee flexions, lower (Moreau et al., 2008) or no (Eken et al., 2013; Moreau et al., 2016) difference was observed between the two groups. In (Eken et al., 2013), lower fatigue in the group with CP also emerged from the analyses of EMG spectra, where individuals with CP showed a smaller decline in the EMG median frequency in the quadriceps and the hamstring muscles.

**Table 2:** Details and results of studies for “body functions and structures”: maximal fatiguing tasks.

**Age (years, mean ± SD) CP – TD**	**Number of participants CP - TD**	**GMFCS**	**Muscles**	**Fatiguing task**	**Outcome measurement**	**Results**	**Conclusions**	**References**
			KE	50 maximal concentric KE and KF contractions at 60°/s with an isokinetic dynamometer	Change in torque during a fatiguing task	Lower decline in CP		
17.2 ± 4.3 17.3 ± 5.0	9 11	I-II-III	KF			Similar decline	Less fatigue in CP	(Moreau et al., 2016)
			RF VM BF		Change in EMG-amplitude during fatiguing task	Similar decline		
			KE		Change of torque during fatiguing task	Lower decline in CP		
			KF			Similar decline		
8.1 ± 2.0 10.4 ± 2.0	7 9	I-II	RF VL VM	35 maximal concentric KE and KF contractions at 60°/s with an isokinetic dynamometer	Change in EMG median frequency during fatiguing task	Lower decline in CP in the RF and VL. Similar decline in two groups in the VM	Less fatigue in CP	(Eken et al., 2013)
			BF ST			Similar decline		
17.5 ± 5.0 16.6 ± 4.5	18 16	I-II-III	KE KF	35 maximal concentric KE and KF contractions at 60°/s with an isokinetic dynamometer	Change in torque during fatiguing task	Lower decline in CP	Less fatigue in CP	(Moreau et al., 2008)
20.5 ± 4.7 20.4 ± 4.5	10 10	I-II-III	PF	Four 30-second maximal isometric plantar flexions with additional M-wave electrical stimulation at supramaximal intensity, interspaced by a resting period of 2–3 s	Change in torque during MVC force before and after the fatiguing task	No change in CP, decrease in TD	Less fatigue in CP	(Neyroud et al., 2017)

In agreement with the above results of lower fatigue in individuals with CP than in TD participants, a greater fatigue resistance was found in the group with CP in comparison to their TD peers in maximal isometric plantar flexions with additional M-wave electrical stimulation at supramaximal intensity (Neyroud et al., 2017).

#### Submaximal Fatiguing Tasks

**Figure 4:**
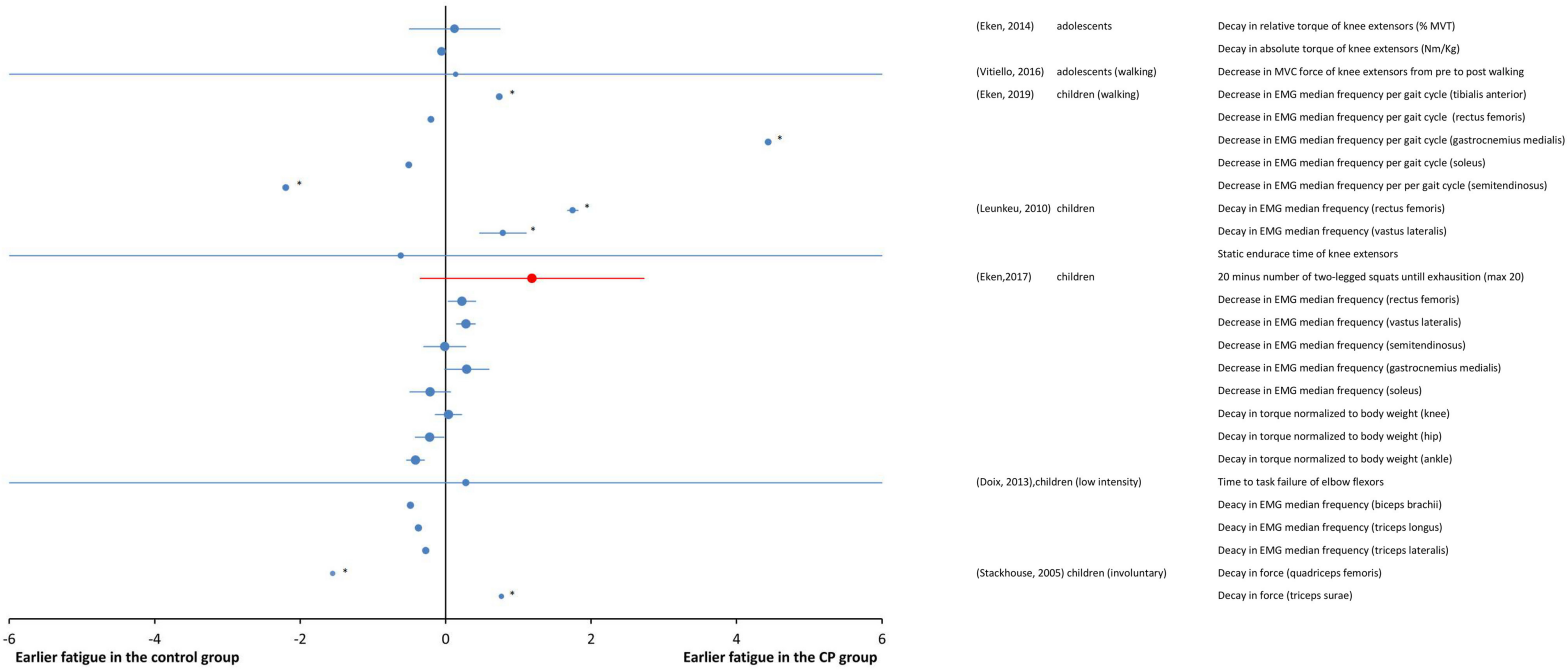
Forest plot of fatigue data in submaximal tasks from measures of “body functions and structures” (blue symbols) and from objective measures of activity (red symbols). The size of the symbols is proportional to the number of participants. The horizontal bars indicate the 95% confidence interval. * indicates statistical significance between the groups (*p* < 0.05).

A total of seven studies at the “body function and structures” level used a submaximal fatiguing task. Data are reported as SMDs in the forest plot of Figure 4. Study details and results are also summarized in Table 3. The participants in some studies (Stackhouse et al., 2005; Leunkeu et al., 2010; Doix et al., 2013; Eken et al., 2017, 2019) were children, whereas adolescents participated in some studies (Eken et al., 2014; Vitiello et al., 2016). The overall effect size was not calculated due to heterogeneity of the data in terms of participant characteristics, the muscles under investigation and the type of fatiguing task.

**Table 3:** Details and results of studies for “body functions and structures”: submaximal fatiguing tasks.

**Age (years, mean ± *SD*) CP - CG**	**Number of participants CP - CG**	**GMFCS**	**Muscles**	**Fatiguing task**	**Outcome measurements**	**Results**	**Conclusions**	**References**
					Change in EMG median frequency during a fatiguing task	Larger decline in CP		
13.0 ± 2.0 14.0 ± 1.0	12 12	I-II	RF VL	Isometric KE contractions at 50% of MVC, maintained as long as possible, with isokinetic device	Change in EMG-amplitude during a fatiguing task	Lower increase in CP	More fatigue in CP	(Leunkeu et al., 2010)
			KE		Static endurance time	Similar isometric endurance time		
			KE		Change in torque during a fatiguing task.	Larger decline in CP		
15.0 ± 9.0 15.0 ± 11.0	16 18	I-II	RT VL VM	Repeated concentric KE contractions until exhaustion with a load at 50–90% of MVC at 60°/s with an isokinetic dynamometer	Change in EMG median frequency during a fatiguing task	Similar decline	More fatigue in CP	(Eken et al., 2014)
					Change in EMG-amplitude during a fatiguing task	Similar increase		
			TA GM S		Change in EMG median frequency. During a fatiguing task	Larger decline in CP		
11.4 ± 3.8 9.1 ± 3.8	13 14	I-II-III	RF ST	Walking Test for 5 min at a self-selected speed		No change in either groups	More fatigue in CP	(Eken et al., 2019)
			TA GM S		Change in EMG-amplitude during a fatiguing task	Larger increase in CP		
			RF ST			No change in either groups		
14.1 ± 1.7 14.1 ± 1.9	10 10	I-II	KE	15-min walk on a treadmill at self-selected speed	MVC before and after a fatigue task with a dynamometer	Significant strength loss in CP, but not in TD	More fatigue in CP	(Vitiello et al., 2016)
			KE KF PF		Change in torque during a fatiguing task	Similar decline		
			RF VL		Change in EMG median frequency during a fatiguing task	Larger decline in CP		
11.9 11.6	20 16	I-II-III	ST	Squats until exhaustion (≤20)		Similar decline	More fatigue in CP	(Eken et al., 2017)
			GM S			No change in either groups		
			RF VL ST		Change in EMG-amplitude during a fatiguing task	Lower increase in CP		
			GM S			Increase in TD, no change in CP		
12.3 ± 2.9 12.4 ± 2.8	15 17	-	BB TB	Isometric elbow flexion contractions until exhaustion, at 25% of MVC with a dynamometer	Change in EMG median frequency during a fatiguing task	Similar decline	Similar fatigue in the two groups	(Doix et al., 2013)
					Change in EMG-amplitude during a fatiguing task	Lower increase in CP		
					Time to task failure	Similar endurance		
10.5 ± 1.9 9.8 ± 1.5	11 10	I-II-III	KE	180 electrically stimulated (involuntary) contractions, at 40% of maximum (plateau) intensity		Lower decline in C	CP showed less fatigue for KE, but similar fatigue for PF compared to TD	(Stackhouse et al., 2005)
			PF		Change in torque during a fatiguing task	Similar decline		

Unlike maximal fatiguing tasks, where decrease in EMG amplitude was a direct measure of fatigue, EMG amplitude changes in submaximal fatiguing tasks reflected the mechanisms to compensate for fatigue. Thereby, the indications given by the EMG amplitude must be carefully evaluated in relation to the fatiguing task as they can have different, sometimes divergent, interpretations. For this reason, the parameter was not included in the forest plot of Figure 4.

In Leunkeu et al. (2010), fatigue, as measured from the decline in median EMG frequency, was greater in individuals with CP than in the TD participants during submaximal isometric contractions of the quadriceps muscle. The increase in EMG amplitude was lower in the group with CP. In Eken et al. (2014), larger fatigue in the group with CP was measured as a larger decrease in peak torque per repetition maximum in a repetitions-to-fatigue protocol of isotonic contractions, however, the EMG responses did not differ between the CP and control group.

EMG was used to measure fatigue during self-paced walking in individuals with CP having mild-to-moderate severity of disability, specifically those with GMFCS levels from I to III (Eken et al., 2019). The EMG data indicated that the group with CP experienced larger fatigue than the TD participants, though selectively in terms of muscle groups and most/least affected leg. Moreover, data also indicated that muscle fatigue in participants with CP during walking occurs prominently in lower leg muscles, and not the upper leg muscles. In Vitiello et al. (2016), using a fatiguing task of 15-min walk on a treadmill at preferred speed, the maximum voluntary contraction (MVC) force decreased significantly from pre- to post- walking in the individuals with CP, while it was preserved in the TD participants, indicating larger fatigue in individuals with CP. In another study (Eken et al., 2017), using a task of squat tests performed until exhaustion (max 20 squats), the results of measurements of decrease in EMG median frequency and increase in EMG amplitude in children with CP and TD children were muscle group dependent. More specifically, a larger decline in EMG median frequency for individuals with CP was observed for quadriceps muscles, while for calf muscles, no changes in EMG median frequency were observed in either groups. In this study, although median EMG frequency data indicated more fatigability in individuals with CP for quadriceps muscles, fatigue measured as the change in net torque during a single squat was not significantly different between groups.

Disagreement from these results of larger fatigue in groups with CP than in control groups were found for very low intensity tasks, where similar levels of fatigue in groups with CP and control groups were observed (Doix et al., 2013), and for fatiguing tasks of involuntary contractions, where lower fatigability was found in the group with CP (Stackhouse et al., 2005). It was found that the groups of children with CP and TD children responded similarly in terms of time to task failure during low force (10–35% of MVT) contractions (Doix et al., 2013). As for the EMG signal of biceps brachii and triceps brachii muscles, the decrease of the EMG median frequency was also similar for the two groups. These results indicate that during sustained low force contractions, children with CP exhibited similar levels of fatigue as children with TD. In Stackhouse et al. (2005), electrically elicited submaximal contractions were used to measure fatigability as force decay. The quadriceps femoris muscle was significantly less fatigable in children with CP than in those with TD, while similar fatigue levels were measured in the triceps surae of both groups.

### Outcome Measures at “Capacity” Level

A total of 4 studies were included at the “capacity” level, out of which 2 are described separately in section outcome measures at “body function and structures” levels and “capacity” levels in maximal fatiguing tasks in highly trained athletes. The two studies included in this section involve submaximal tasks and their details and results are summarized in Table 4.

**Table 4:** Details and results of studies for “capacity”: submaximal fatiguing tasks.

**Age (years, mean ± *SD*) CP - CG**	**Number of participants CP - CG**	**GMFCS**	**Investigated muscles**	**Fatiguing task**	**Outcome measurements**	**Results**	**Conclusions**	**References**
11.9 11.6	20 16	I-II-III	Whole-body with focus on lower limbs	Squats until exhaustion (≤20)	Number of squats	Lower endurance in squat test in CP children	More fatigue in CP	(Eken et al., 2017)
14.1 ± 1.7 14.1 ± 1.9	10 10	I-II	-	15-min walk on a treadmill at self-selected speed	Pictorial Children's Effort Rating Table (0–10) score before and after the fatiguing task	Larger median value in CP (somewhat strong) than TD (weak/light)	More fatigue in CP	(Vitiello et al., 2016)

Two studies at the “capacity” level used submaximal tasks. As these studies also used the outcome measures of the “body function and structures” level, their results are all together reported as SMDs in the forest plot of Figure 4. In one study (Eken et al., 2017), using a fatiguing task performed until exhaustion (max 20 squats), the resistance was significantly greater in the TD participants than in the individuals with CP. In the other study (Vitiello et al., 2016), the subjects performed a 15-min walk on a treadmill at preferred speed; after the fatiguing test, the individuals with CP reported a higher perception of effort as compared to the TD participants in the Pictorial Children's Effort Rating Table (0–10) (Marinov et al., 2008).

### Outcome Measures at “Body Function and Structures” Level and “Capacity” Level in Maximal Fatiguing Tasks in Highly Trained Athletes

**Figure 5:**
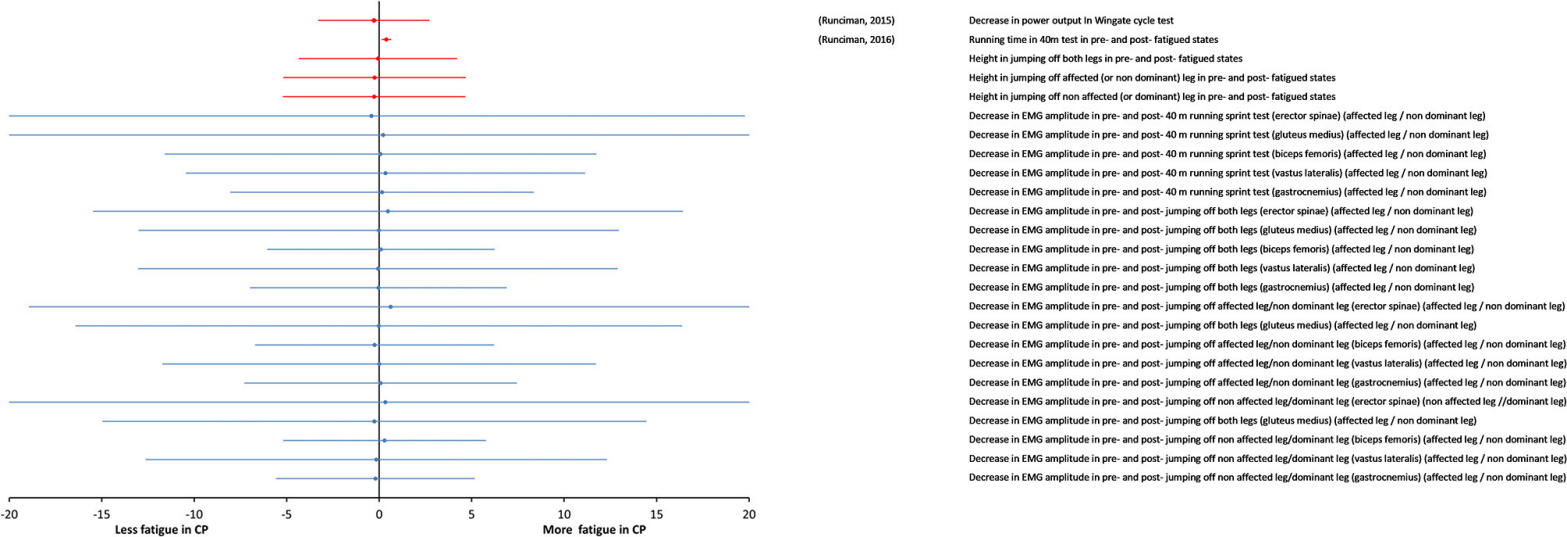
Forest plot of fatigue data in maximal tasks in highly trained athletes (Paralympic athletes). Data from measures of “body functions and structures” (blue symbols) and from objective measures of activity (red symbols). The size of the symbols is proportional to the number of participants. The horizontal bars indicate the 95% confidence interval. * indicates statistical significance between the groups (*p* < 0.05).

In this section, we separately consider the studies carried out on highly trained athletes with CP and TD athletes. The details and results of these studies are summarized in Table 5. Strenuous protocols of functional tasks were used in these studies. These studies are noteworthy in particular, because they measure fatigue in complex tasks like running, cycling, and jumping, which involve integration of muscle groups. Additionally, they highlight the roles of physical training and muscle weakness, as they involve athletes with CP with significantly less power and strength deficits than typical individuals with CP. In the two studies (Runciman et al., 2015, 2016), strenuous tests of sprint running and jumping, cycling sprint and maximal shuttle running were used and fatigue was measured from the change in performance and EMG activity in pre-fatigue and post-fatigue states. The key finding of all these studies was that fatigue measured by both, the change in performance and the EMG amplitude was similar for the groups with CP and control groups of well-trained athletes, even though performance itself was significantly impaired in the groups with CP. This result is clearly visible in the forest plot of Figure 5. The overall effect size was not calculated, because it is very likely that the participants in these two studies were the same individuals.

**Table 5:** Details and results of studies for “body functions and structures” and “capacity”: maximal fatiguing tasks in highly trained athletes.

**Age (years, mean ± *SD*) CP - CG**	**Number of participants CP - CG**	**GMFCS**	**Muscles**	**Fatiguing task**	**Outcome measurements**	**Results**	**Conclusions**	**References**
21.6 ± 4.2 23.4 ± 3.0	5 paralympic athletes 16 able-bodied athletes	I-II-III	ES GT VL BF GM	30-s Wingate anaerobic power test on cycle ergometer	Change in EMG-amplitude and EMG median frequency during a fatiguing task	Similar decline Similar performance decline	Similar fatigue in the two groups	(Runciman et al., 2015)
			Whole-body with focus on lower limbs		Change in power output during a fatiguing task.			
			ES GT VL BF GM	Multistage shuttle run test to exhaustion	Change in EMG-amplitude during 40 m sprint test and vertical jump before and after a fatiguing task	Similar decline	Similar fatigue in the two groups	
22.7 ± 3.6 26.1 ± 3.5	6 paralympic athletes 13 able-bodied athletes	I	Whole-body with focus on lower limbs		Time to performance 40 m sprint test before and after the fatiguing task	Similar performance decline	Similar fatigue in 40 m sprint test. No fatigue in vertical jump in the the two groups	(Runciman et al., 2016)
					Vertical jump height before and after the fatiguing task	The performance in vertical jump did not change in either groups		

### Outcome Measures at “Performance” Level and at “Participation” Level

**Figure 6:**
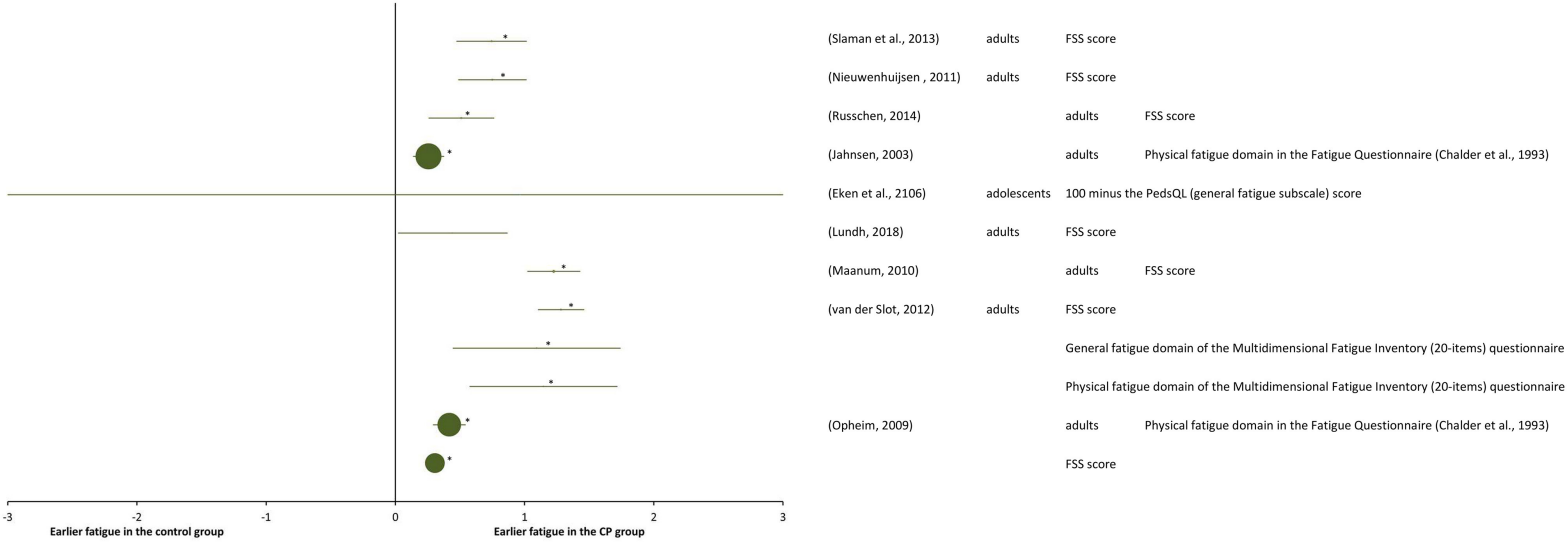
Forest plot of data of self-perceived fatigue in daily physical activity from activity “performance and/or participation” measures. The size of the symbols is proportional to the number of participants. The horizontal bars indicate the 95% confidence interval. * indicates statistical significance between the groups (*p* < 0.05).

A total of nine studies were included at the “performance” level and at the “participation” level (Table 6). They are reported as SMDs in the forest plot of Figure 6. The study details and results are summarized in Table 6. Significant statistical heterogeneity of SMD data (*p* = 0.012) was obtained with the Cochran Q test and substantial heterogeneity (81%) was obtained with the *I*^2^ test, therefore, the overall effect size was not calculated.

**Table 6:** Details and results of studies for “performance” and/or “participation”: self-reported fatigue in daily physical activity.

**Age (years, mean ± *SD*) CP - TD**	**Number of participant CP – TD**	**GMFCS**	**Outcome measurements**	**Results**	**Conclusion**	**References CP – TD**
36.4 ± 5.8 54.2 ± 14.8	42 113	I-II-III	FSS	The mean score of the group with CP was 4.1 ± 1.3 as compared to 2.9 ± 1.1 of a TD sample of literature. Fifty percent of the group with CP did not experience fatigue (score <4.0), 31% were fatigued (score > 4.0 and <5.1) and another 19% were severely fatigued (score ≥ 5.1).	More fatigue in CP	(Merkies et al., 1999; Nieuwenhuijsen et al., 2011)
36.0 ± 6.0 54.2 ± 14.8	36 113	I-II-III	FSS	The mean score of the group with CP was 4.1 ± 1.3 as compared to 2.9 ± 1.1 of a TD sample of literature. Eighteen percent of all individuals with CP were fatigued (score > 4.0 and <5.1) and 30% were severely fatigued (score ≥ 5.1).	More fatigue in CP	(Merkies et al., 1999; Slaman et al., 2013)
20.0 ± 2.8 47.0 ± 18.0	50 454	I-II-III-IV	FSS	The mean score of the group with CP was 3.8 ± 1.8 as compared to 3.0 ± 1.08 of a TD sample of literature. Fatigue was higher in the unilateral group than in the bilateral group	More fatigue in CP	(Valko et al., 2008; Lundh et al., 2018)
20.0 ± 2.8 54.2 ± 14.8	56 113	I-II-III	FSS	The mean score of the group with CP was 3.7 ± 1.4 as compared to 3.0 ± 1.08 of a TD sample of literature. 39.3% of all individuals with CP were fatigued (score > 4.0) and 12.5% were severely fatigued (score ≥ 5.1) Participants with bilateral CP were more fatigued compared to those with unilateral CP.	More fatigue in CP	(Merkies et al., 1999; Russchen et al., 2014)
34.0 ± 11.0 45.0 ± 17.0	406 2,323	-	Questionnaire for physical and mental fatigue	CP reported more physical, but not more mental fatigue, than the TD sample of literature. CP with moderate grade of functional abilities had higher prevalence of fatigue than participants with mild or severe grades	More fatigue in CP	(Loge et al., 1998; Jahnsen et al., 2003)
15.4 ± 2.3	17 18	I-II	1) PedsQL 2) Life-Habits questionnaire	Group with CP reported higher scores on fatigue scales than TD.	More fatigue in CP	(Eken et al., 2016)
40.4 ± 10.6 45.2 ± 26.0 45.0 ± 17.0	149 1,893 2,323	I-II-III-IV	1) FSS 2) Fatigue Questionnaire	Fatigue scores of both questionnaires were higher than scores of TD sample of literature. Unlike the physical subscale, in the mental subscale, there was no difference between groups	More fatigue in CP	(Loge et al., 1998; Lerdal et al., 2005; Opheim et al., 2009)
36.4 ± 5.8 54.2 ± 14.8 41.4 ± 1.3	56 113 67	I-II-III-IV	1) FSS 2) MFI-20	Fatigue scores of both questionnaires were higher than the scores of TD sample of literature. In the group with CP, 20% individuals were fatigued and 41% were severely fatigued.	More fatigue in CP	(Merkies et al., 1999; Minderhoud et al., 2003; Van der Slot et al., 2012)
39.0 ± 12.0 54.2 ± 14.8	126 113	I-II-III	FSS	The average CP score was 4.8 ± 1.4, which corresponds to severe fatigue and is higher than that of the TD sample of literature.	More fatigue in CP	(Merkies et al., 1999; Maanum et al., 2010)

*TD, typically developing/developed control; KE, knee extensors; KF, knee flexors; RF, rectus femoris; VL, vastus lateralis; VM, vastus medialis; BF, biceps femoris; ST, semitendinous; PF, plantar flexor; MVC, maximum voluntary contractions; TA, tibialis anterior; GM, gastrocnemius medialis; S, soleus; BB, biceps brachii; TB, triceps brachii; ES, erector spinae; GM, gastrocnemius medius; GT, gluteus medius; GMFCS, Gross Motor Function Classification System; PedsQL, Pediatric Quality of Life Inventory Multidimensional Fatigue Scale; MFI-20, Multidimensional Fatigue Inventory; FSS, Fatigue Severity Scale*.

Among all the selected studies that focused on self-reported fatigue in performing daily physical activities in adolescents and adults with CP, it turned out that the groups with CP experienced higher fatigue than the control groups of general population, which was in agreement with measurements of objective-fatigue in submaximal tasks. One of the first studies on self-perceived fatigue (Jahnsen et al., 2003), reported prevalence of self-perceived fatigue in adults with CP when compared with a Norwegian normative sample of literature (Loge et al., 1998), as measured with a multidimensional questionnaire, containing among other instruments the Fatigue Questionnaire (Chalder et al., 1993). Adults with CP reported significantly more physical, but not more mental fatigue, than the general population. The 9-item Fatigue Severity Scale questionnaire (FSS), assessing self-perceived fatigue on a scale from 1 to 7, was used in a number of successive studies on adults with CP (Opheim et al., 2009; Maanum et al., 2010; Nieuwenhuijsen et al., 2011; Van der Slot et al., 2012; Slaman et al., 2013; Russchen et al., 2014; Lundh et al., 2018). In several studies (Maanum et al., 2010; Nieuwenhuijsen et al., 2011; Slaman et al., 2013; Russchen et al., 2014), self-perceived fatigue scores of adults with CP were compared with the normative score (2.85) of Dutch TD adults (Merkies et al., 1999). With this normative control, FSS scores larger than 1 SD (standard deviation) above the mean normative score for TD individuals indicated fatigue, while scores larger than 2 SD indicated severed fatigue. In one study (Nieuwenhuijsen et al., 2011), half of the 42 individuals with CP were reported to be experiencing fatigue, among these 19% were experiencing severe fatigue, in another study (Slaman et al., 2013), 18% of the 36 individuals with CP experienced fatigue and 30% severe fatigue, and in another one (Russchen et al., 2014) 40% of the 56 individuals with CP experienced fatigue, including 12% experiencing severe fatigue. Larger fatigue in the individuals with CP than in control group was obtained also in the studies (Maanum et al., 2010; Lundh et al., 2018).

Other researchers reporting combined results of multiple fatigue questionnaires obtained very similar results as those mentioned above, with significantly higher fatigue for the group with CP compared to the general population. In a study of self-perceived fatigue in a population of 149 adults with CP (Opheim et al., 2009), both the Fatigue Questionnaire score (Chalder et al., 1993) and the FSS score showed significantly higher fatigue for the group with CP compared to general population data from the literature (Loge et al., 1998; Lerdal et al., 2005). In another study (Van der Slot et al., 2012), fatigue of 56 adults with spastic bilateral CP was measured using the FSS and the Multidimensional Fatigue Inventory (MFI) (20-items) (Smets et al., 1996) questionnaires and resulted to be significantly higher in these individuals with CP than in general population data from literature (Merkies et al., 1999; Minderhoud et al., 2003).

The results of another study (Russchen et al., 2014) indicated that fatigue is already present at a relatively young age amongst adults with CP. Indeed, the same result of larger fatigue in individuals with CP was also found in adolescents with CP (Eken et al., 2016) as compared to TD controls, using the Pediatric Quality of Life Inventory Multidimensional Fatigue Scale (Varni et al., 2004).

## Discussion

The results provided by the studies carried out with maximal fatiguing tasks at “body function and structures” level demonstrated less fatigue in participants with CP vs. TD controls. In studies on highly trained individuals with CP, fatigue was similar in the group with CP as compared to the controls, even if performances themselves were significantly compromised in individuals with CP. Conversely, when sub-maximal fatiguing tasks were used at all ICF levels, the results showed larger fatigue in individuals with CP than in TD controls.

### Fatigue in Maximal Tasks at “Body Function and Structures” Level

Fatigue during maximal tasks from “body functions and structures” measures, which was assessed as decay of peak torque or shift of EMG median frequency to lower values turned out to be larger in the control groups than in the groups with CP. However, it is essential to consider that individuals with CP are affected by UMNS; therefore, they have a functional damage to the descending motor pathways (Rosenbaum et al., 2007a). This functional deficit caused by reduction of the synaptic input that the upper motor neurons provide to spinal motor neurons, is probably able to limit the ability of individuals with CP to activate the largest spinal motor neurons (Stackhouse et al., 2005; Leunkeu et al., 2010; Eken et al., 2013). Hence, according to the size principle of Henneman et al. (1965), a high synaptic input is required to activate large spinal motor neurons, while lower synaptic inputs are sufficient to activate the smaller ones. It is therefore likely that individuals with CP may have difficulty in activating the largest spinal motor neurons i.e., the innervating fast fatigable (type IIB) muscle fibers. This difficulty in activating fast fatigable muscle fibers could explain why individuals with CP show less fatigue than TD controls when a maximal fatiguing task is used. It can be concluded that in carrying out the maximal fatiguing motor tasks, the individual with CP develops a smaller force output than that produced by a TD person. However, this force output is less susceptible to fatigue (Moreau et al., 2008, 2016; Eken et al., 2013; Neyroud et al., 2017).

Interestingly, when the maximal fatiguing tasks were used in case of highly trained individuals with CP and TD athletes (Runciman et al., 2015, 2016), fatigue was found to be similar in the two groups. As suggested by the authors of these studies, it is possible that highest level of training given to these individuals with CP over many years, helped them adapt to normal physiology and improved their ability to recruit the fast fatigable fibers. However, it is important to consider that the participants in these studies were selected carefully. They were highly talented. Despite their disability, they were able to run, jump, and cycle.

### Fatigue in Submaximal Tasks at All ICF Levels

The literature for submaximal fatiguing tasks is varied, including measures such as EMG measurements and torque, as well as objective activity measures such as assessment of fatigue in functional tasks in laboratory environment and during the execution of daily living activities.

Using outcome measures at the “body functions and structures” and “activity” levels with submaximal fatiguing tasks, higher fatigue resulted in individuals with CP than in TD controls. This finding appears even more consistent and robust if it is considered that larger fatigue in the group with CP was reported in all the studies in which fatigue was subjectively assessed using outcome measures at the levels of “performance” and/or “participation.” These studies assessed subjective fatigue in an environmental context, during the execution of daily living activities. Obviously, as daily living activities are submaximal motor tasks, the consistency of the results is explained.

The reduced ability of individuals with CP to activate the compensatory mechanisms for maintaining a given task may be a factor that contributes to their increased fatigue (Leunkeu et al., 2010; Doix et al., 2013; Eken et al., 2017). This reduced ability can be seen from the changes in EMG amplitude during fatiguing tasks. Changes in EMG amplitude during submaximal tasks reflect two phenomena: the degree of fatigue induced by the task and the participant's ability to implement the compensatory strategy. When the fatiguing task was the same in participants with CP and TD participants (for instance walking for 5 min), it induced fatigue only in participants with CP or induced more fatigue in them as compared to the TD individuals. In this case, EMG amplitude increased only in participants with CP or increased more in them than in TD controls (Eken et al., 2019). When the fatiguing task was tailored according to the participant's potential (for instance, a submaximal motor task until exhaustion), the EMG amplitude showed a lower increase in participants with CP, revealing their reduced ability to activate the compensatory mechanism for maintaining the task and their consequent increased fatigue. As far as submaximal fatiguing tasks are considered, it is conceivable that, in carrying out the task, both participants with CP and TD participants first activate the slow oxidative muscle fibers. The participants of both groups (CP and TD controls) would, therefore, activate the same type of muscle fibers. In carrying out the submaximal fatiguing task, the large motor neurons, which innervate the fast fatigable muscle fibers, are likely to be recruited at a later stage, in order to temporarily counteract fatigue. The inability to recruit the large spinal motor neurons, discussed above, could be one of the factors causing fatigue to be greater in participants with CP. Only one study performed with submaximal contractions revealed less fatigue or similar fatigue in individuals with CP as compared to the TD controls (Stackhouse et al., 2005). This was the only study in which muscle contractions were obtained by electrical stimulation of the peripheral nerve. This result is likely to depend on the higher proportion of slow oxidative muscle fibers in the muscles of individuals with CP (Marbini et al., 2002), due to the atrophy of the fast fatigable fibers (Castle et al., 1979). The discrepancy of the results obtained with submaximal voluntary and peripherally-triggered muscle contractions, probably indicates a central origin, thereby, explaining the increased fatigue in individuals with CP.

In conclusion, the apparent inconsistency in literature results on fatigue in individuals with CP compared to that in TD people can be explained in terms of specific protocol of the fatiguing tasks, but not in terms of the different types of measured outcomes. In fact, fatigue data measured in participants with CP and control participants were task-dependent. During maximum fatiguing tasks in measures at the “body function and structures” level, a lower level of fatigue was found in individuals with CP compared to TD participants, possibly due to the inability of individuals with CP to recruit highly fatigable muscle fibers, owing to the neurological origin of this disorder. On the other hand, during the submaximal fatiguing tasks in measures at the “body function and structures and activity” levels, the group with CP experienced larger fatigue than the control groups, likely due to their inability to develop neurophysiological strategies to compensate for fatigue. Self-perceived fatigue in daily physical activities in measures at the “performance and participation” levels was larger in individuals with CP than in control participants in all selected studies; in a hypothesis that classifies daily physical activity as a submaximal task, this result is consistent with the framework, as the specific protocol of the fatiguing task is predominant in determining fatigue in individuals with CP.

The results of the included studies belonging to all ICF levels should be confirmed by further studies with a larger number of participants, in order to clarify the different aspects of fatigue and the underlying mechanisms in relation to the specific protocol of the fatiguing tasks used.

## Perspectives

The literature data collected in this review reported larger muscle fatigue in individuals with CP than in TD participants, during tasks in standardized environments and in daily activities. These results could provide a framework for future studies in order to gain an insight on the underlying physiological mechanisms at play, thereby outlining rehabilitation methodologies, accordingly. It appears that tailored rehabilitation programs for individuals with CP could be particularly effective if they specifically target the preservation of fast muscle fibers against atrophy, improvement of recruitment capacity of fast fatigue muscle fibers, and improvement of synchronization capacity of the recruited motor units.

## Data Availability Statement

The original contributions presented in the study are included in the article/supplementary material, further inquiries can be directed to the corresponding author/s.

## Author Contributions

LP, IP, TI, and CT contributed to study design/planning. LP and IP contributed to data collection/entry. LP, IP, and CT contributed to data analysis/statistics and literature analysis/search. LM and CT contributed to data interpretation and collection of funds. All the authors contributed to preparation of the manuscript.

## Conflict of Interest

The authors declare that the research was conducted in the absence of any commercial or financial relationships that could be construed as a potential conflict of interest.

## References

[B1] Allen D. G. Lamb G. D. Westerblad H. (2008). Skeletal Muscle Fatigue: Cellular Mechanisms. Physiological Reviews, 88, 287–332. 10.1152/physrev.00015.2007 18195089

[B2] Battistella L. R. Moran de Brito C. M. (2002). International classification of functioning disability and health (ICF). Acta Fisiátrica 9, 98–101. 10.5935/0104-7795.20020003

[B3] Bigland-Ritchie B. Johansson R. Lippold O. C. Woods J. J. (1983). Contractile speed and EMG changes during fatigue of sustained maximal voluntary contractions. J. Neurophysiol. 50, 313–324. 10.1152/jn.1983.50.1.313 6308182

[B4] Castle M. E. Reyman T. A. Schneider M. (1979). Pathology of spastic muscle in cerebral palsy. Clin. Orthopaed. Relat. Res. 142, 223–232. 10.1097/00003086-197907000-00036 159152

[B5] Chalder T. Berelowitz G. Pawlikowska T. Watts L. Wessely S. Wright D.. (1993). Development of a fatigue scale. J. Psychosomatic Res. 37, 147–153. 10.1016/0022-3999(93)90081-P 8463991

[B6] Doix A.-C. Anette Gulliksen M. Braendvik S. M. Roeleveld K. (2013). Fatigue and muscle activation during submaximal elbow flexion in children with cerebral palsy. J. Electromyogr. Kinesiol. 23, 721–726. 10.1016/j.jelekin.2012.12.005 23375713

[B7] Eken M. M. Braendvik S. M. Bardal E. M. Houdijk H. Dallmeijer A. J. Roeleveld K. (2019). Lower limb muscle fatigue during walking in children with cerebral palsy. Dev. Med. Child Neurol. 61, 212–218. 10.1111/dmcn.14002 30156008 PMC7379556

[B8] Eken M. M. Dallmeijer A. J. Doorenbosch C. A. M. Dekkers H. Becher J. G. Houdijk H.. (2014). Assessment of muscle endurance of the knee extensor muscles in adolescents with spastic cerebral palsy using a submaximal repetitions-to-fatigue protocol. Archiv. Phys. Med. Rehabil. 95, 1888–1894. 10.1016/j.apmr.2014.05.010 25183298

[B9] Eken M. M. Dallmeijer A. J. Houdijk H. Doorenbosch C. A. M. (2013). Muscle fatigue during repetitive voluntary contractions: a comparison between children with cerebral palsy, typically developing children and young healthy adults. Gait Posture 38, 962–967. 10.1016/j.gaitpost.2013.05.004 23810336

[B10] Eken M. M. Harlaar J. Dallmeijer A. J. de Waard E. van Bennekom C. A. M. Houdijk H. (2017). Squat test performance and execution in children with and without cerebral palsy. Clin. Biomech. 41, 98–105. 10.1016/j.clinbiomech.2016.12.006 28040656

[B11] Eken M. M. Houdijk H. Doorenbosch C. A. M. Kiezebrink F. E. M. van Bennekom C. A. M. Dallmeijer A. J. (2016). Relations between muscle endurance and subjectively reported fatigue, walking capacity, and participation in mildly affected adolescents with cerebral palsy. Dev. Med. Child Neurol. 58, 814–821. 10.1111/dmcn.13083 26915305

[B12] Gandevia S. C. (2001). Spinal and supraspinal factors in human muscle fatigue. Physiol. Rev. 81, 1725–1789. 10.1152/physrev.2001.81.4.1725 11581501

[B13] Henneman E. Somjen G. Carpenter D. O. (1965). Excitability and inhibitability of motoneurons of different sizes. J. Neurophysiol. 28, 599–620. 10.1152/jn.1965.28.3.599 5835487

[B14] Higgins J. P. T. Thompson S. G. (2002). Quantifying heterogeneity in a meta-analysis. Statist. Med. 21, 1539–1558. 10.1002/sim.1186 12111919

[B15] Jahnsen R. Villien L. Stanghelle J. K. Holm I. (2003). Fatigue in adults with cerebral palsy in norway compared with the general population. Dev. Med. Child Neurol. 45, 296–303. 10.1111/j.1469-8749.2003.tb00399.x 12729142

[B16] JBI (2020). Critical Appraisal Tools. Joanna Briggs Institute. Available online at: https://joannabriggs.org/critical-appraisal-tools (accessed September, 2019).

[B17] Kaasa S. Loge J. H. Knobel H. Jordhoy M. S. Brenne E. (1999). Fatigue. Measures and relation to pain. Acta Anaesthesiol. Scand. 43, 939–947. 10.1034/j.1399-6576.1999.430911.x 10522741

[B18] Kirkendall D. T. (1990). Mechanisms of peripheral fatigue. Med. Sci. Sports Exerc. 22, 444–449. 10.1249/00005768-199008000-00004 2205780

[B19] Komi P. V. Tesch P. (1979). EMG frequency spectrum, muscle structure, and fatigue during dynamic contractions in man. Eur. J. Appl. Physiol. Occup. Physiol. 42, 41–50. 10.1007/BF00421103 499196

[B20] Lerdal A. Wahl A. Rustoen T. Hanestad B. R. Moum T. (2005). Fatigue in the general population: a translation and test of the psychometric properties of the norwegian version of the fatigue severity scale. Scand. J. Public Health 33, 123–130. 10.1080/14034940410028406 15823973

[B21] Leunkeu A. N. Keefer D. J. Imed M. Ahmaidi S. (2010). Electromyographic (EMG) analysis of quadriceps muscle fatigue in children with cerebral palsy during a sustained isometric contraction. J. Child Neurol. 25, 287–293. 10.1177/0883073809338734 19794102

[B22] Loge J. H. Ekeberg O. Kaasa S. (1998). Fatigue in the general norwegian population: normative data and associations. J. Psychosomat. Res. 45, 53–65. 10.1016/S0022-3999(97)00291-2 9720855

[B23] Lundh S. Nasic S. Riad J. (2018). Fatigue, quality of life and walking ability in adults with cerebral palsy. Gait Posture 61, 1–6. 10.1016/j.gaitpost.2017.12.017 29277025

[B24] Maanum G. Jahnsen R. Froslie K. F. Larsen K. L. Keller A. (2010). Walking ability and predictors of performance on the 6-minute walk test in adults with spastic cerebral palsy. Dev. Med. Child Neurol. 52, e126–e132. 10.1111/j.1469-8749.2010.03614.x 20163429

[B25] Marbini A. Ferrari A. Cioni G. Bellanova M. F. Fusco C. Gemignani F. (2002). Immunohistochemical study of muscle biopsy in children with cerebral palsy. Brain Dev. 24, 63–66. 10.1016/S0387-7604(01)00394-1 11891093

[B26] Marinov B. Mandadjieva S. Kostianev S. (2008). Pictorial and verbal category-ratio scales for effort estimation in children. Child. 34, 35–43. 10.1111/j.1365-2214.2007.00767.x 18171442

[B27] Merkies I. S. Schmitz P. I. Samijn J. P. van der Meche F. G. van Doorn P. A. (1999). Fatigue in immune-mediated polyneuropathies. European Inflammatory Neuropathy Cause and Treatment (INCAT) Group. Neurology 53, 1648–1654. 10.1212/WNL.53.8.1648 10563607

[B28] Merletti R. Rainoldi A. Farina D. (2004). Myoelectric manifestations of muscle fatigue, in Electromyography, ed Merletti R. Parker F. A. (Hoboken, NJ: IEEE Press). 10.1002/0471678384.ch9

[B29] Minderhoud I. M. Oldenburg B. van Dam P. S. van Berge Henegouwen G. P. (2003). High prevalence of fatigue in quiescent inflammatory bowel disease is not related to adrenocortical insufficiency. Am. J. Gastroenterol. 98, 1088–1093. 10.1111/j.1572-0241.2003.07414.x 12809832

[B30] Moola K. Munn Z. Tufanaru C. Sears K. Sftec R. Currie M.. (2020). Chapter 7: Systematic Reviews of Etiology and Risk - JBI Manual for Evidence Synthesis - JBI GLOBAL WIKI. JBI Manual for Evidence Synthesis. Available online at: https://wiki.jbi.global/display/MANUAL/Chapter+7%3A+Systematic+reviews+of+etiology+and+risk (accessed September, 2019).

[B31] Moreau N. G. Heather K. Olson M. W. (2016). A potential mechanism by which torque output is preserved in cerebral palsy during fatiguing contractions of the knee extensors. Muscle Nerve 53, 297–303. 10.1002/mus.24735 26095979

[B32] Moreau N. G. Li L. Geaghan J. P. Damiano D. L. (2008). Fatigue resistance during a voluntary performance task is associated with lower levels of mobility in cerebral palsy. Archiv. Phys. Med. Rehabil. 89, 2011–2016. 10.1016/j.apmr.2008.03.012 18722588 PMC2668210

[B33] Neyroud D. Armand S. De Coulon G. Dias Da Silva S. R. Maffiuletti N. A. Kayser B.. (2017). Plantar flexor muscle weakness and fatigue in spastic cerebral palsy patients. Res. Dev. Disabil. 61, 66–76. 10.1016/j.ridd.2016.12.015 28064025

[B34] Nieuwenhuijsen C. van der Slot W. M. A. Dallmeijer A. J. Janssens P. J. Stam H. J. Roebroeck M. E.. (2011). Physical fitness, everyday physical activity, and fatigue in ambulatory adults with bilateral spastic cerebral palsy. Scand. J. Med. Sci. Sports 21, 535–542. 10.1111/j.1600-0838.2009.01086.x 20459469

[B35] Opheim A. Jahnsen R. Olsson E. Stanghelle J. K. (2009). Walking function, pain, and fatigue in adults with cerebral palsy: a 7-year follow-up study. Dev. Med. Child Neurol. 51, 381–388. 10.1111/j.1469-8749.2008.03250.x 19207296

[B36] Oskoui M. Coutinho F. Dykeman J. Jette N. Pringsheim T. (2013). An update on the prevalence of cerebral palsy: a systematic review and meta-analysis. Dev. Med. Child Neurol. 55, 509–519. 10.1111/dmcn.12080 23346889

[B37] Rosenbaum P. Paneth N. Leviton A. Goldstein M. Bax M. (2007a). Definition and classification document. Dev. Med. Child Neurol. 49, 8–14. 17370477

[B38] Rosenbaum P. Paneth N. Leviton A. Goldstein M. Bax M. Damiano D.. (2007b). A report: the definition and classification of cerebral palsy April 2006. Dev. Med. Child Neurol. Suppl. 109, 8−14. 17370477

[B39] Runciman P. Derman W. Ferreira S. Albertus-Kajee Y. Tucker R. (2015). A descriptive comparison of sprint cycling performance and neuromuscular characteristics in able-bodied athletes and paralympic athletes with cerebral palsy. Am. J. Phys. Med. Rehabil. 94, 28–37. 10.1097/PHM.0000000000000136 24919082

[B40] Runciman P. Tucker R. Ferreira S. Albertus-Kajee Y. Derman W. A. (2016). Effects of induced volitional fatigue on sprint and jump performance in paralympic athletes with cerebral palsy. Am. J. Phys. Med. Rehabil. 95, 277–290. 10.1097/PHM.0000000000000372 26368834

[B41] Russchen H. A. Slaman J. Stam H. J. van Markus-Doornbosch F. van den Berg-Emons R. J. Roebroeck M. E. (2014). Focus on fatigue amongst young adults with spastic cerebral palsy. J. Neuroeng. Rehabil. 11:161. 10.1186/1743-0003-11-161 25495688 PMC4274699

[B42] Slaman J. Bussmann J. van der Slot W. M. Stam H. J. Roebroeck M. E. van den Berg-Emons R. J. (2013). Physical strain of walking relates to activity level in adults with cerebral palsy. Archiv. Phys. Med. Rehabil. 94, 896–901. 10.1016/j.apmr.2012.11.005 23149309

[B43] Smets E. M. Garssen B. Cull A. de Haes J. C. (1996). Application of the multidimensional fatigue inventory (MFI-20) in cancer patients receiving radiotherapy. Br. J. Cancer 73, 241–245. 10.1038/bjc.1996.42 8546913 PMC2074317

[B44] Stackhouse S. K. Binder-Macleod S. A. Lee S. C. K. (2005). Voluntary muscle activation, contractile properties, and fatigability in children with and without cerebral palsy. Muscle Nerve 31, 594–601. 10.1002/mus.20302 15779003 PMC3069850

[B45] Trompetto C. Curra A. Puce L. Mori L. Serrati C. Fattapposta F.. (2019). Spastic dystonia in stroke subjects: prevalence and features of the neglected phenomenon of the upper motor neuron syndrome. Clin. Neurophysiol. 130, 521–527. 10.1016/j.clinph.2019.01.012 30776732

[B46] Valko P. O. Bassetti C. L. Bloch K. E. Held U. Baumann C. R. (2008). Validation of the fatigue severity scale in a swiss cohort. Sleep 31, 1601–1607. 10.1093/sleep/31.11.1601 19014080 PMC2579971

[B47] Van der Slot W. M. A. Nieuwenhuijsen C. Van Den Berg-Emons R. J. G. Bergen M. P. Hilberink S. R. Stam S. J.. (2012). Chronic pain, fatigue, and depressive symptoms in adults with spastic bilateral cerebral palsy. Dev. Med. Child Neurol. 54, 836–842. 10.1111/j.1469-8749.2012.04371.x 22809436

[B48] Varni J. W. Burwinkle T. M. Katz E. R. Meeske K. Dickinson P. (2004). The PedsQL^TM^ in pediatric cancer: reliability and validity of the pediatric quality of life inventory^TM^ generic core scales, multidimensional fatigue scale, and cancer module. Cancer 94, 2090–2106. 10.1002/cncr.10428 11932914

[B49] Vitiello D. Pochon L. Malatesta D. Girard O. Newman C. J. Degache F. (2016). Walking-induced muscle fatigue impairs postural control in adolescents with unilateral spastic cerebral palsy. Res. Dev. Disabil. 53–54, 11–18. 10.1016/j.ridd.2016.01.019 26851383

